#  New mechanisms for bacterial degradation of sulfoquinovose

**DOI:** 10.1042/BSR20220314

**Published:** 2022-10-21

**Authors:** Yifeng Wei, Yang Tong, Yan Zhang

**Affiliations:** 1 Singapore Institute of Food and Biotechnology Innovation, Agency for Science, Technology and Research (A*STAR), Singapore 138669, Singapore; 2 Tianjin Key Laboratory for Modern Drug Delivery and High-Efficiency, Collaborative Innovation Center of Chemical Science and Engineering, School of Pharmaceutical Science and Technology, Tianjin University, Tianjin 300072, China; 3 Frontiers Science Center for Synthetic Biology (Ministry of Education), Tianjin University, Tianjin 300072, China

**Keywords:** adolase, C-S cleavage, glycyl radical enzyme, mutarotase, sulfoglycolysis, sulfoquinovose

## Abstract

Sulfoquinovose (SQ, 6-deoxy-6-sulfo-D-glucose) is a sulfo-sugar with a ubiquitous distribution in the environment due to its production by plants and other photosynthetic organisms. Bacteria play an important role in degradation of SQ and recycling of its constituent sulfur and carbon. Since its discovery in 1963, SQ was noted to have a structural resemblance to glucose-6-phosphate and proposed to be degraded through a pathway analogous to glycolysis, termed sulfoglycolysis. Studies in recent years have uncovered an unexpectedly diverse array of sulfoglycolytic pathways in different bacteria, including one analogous to the Embden–Meyerhof–Parnas pathway (sulfo-EMP), one analogous to the Entner–Doudoroff pathway (sulfo-ED), and two involving sulfo-sugar cleavage by a transaldolase (sulfo-TAL) and transketolase (sulfo-TK), respectively, analogous to reactions in the pentose phosphate (PP) pathway. In addition, a non-sulfoglycolytic SQ degradation pathway was also reported, involving oxygenolytic C-S cleavage catalyzed by a homolog of alkanesulfonate monooxygenase (sulfo-ASMO). Here, we review the discovery of these new mechanisms of SQ degradation and lessons learnt in the study of new catabolic enzymes and pathways in bacteria.

## Introduction

Sulfoquinovose (SQ) is the polar headgroup of sulfolipids such as sulfiquinovosyldiacylglycerol (SQDG) present in the thylakoid membranes of chloroplasts in plants and other photosynthetic eukaryotes, and in the cytoplasmic membranes of many photosynthetic bacteria [1,2]. The annual global production of SQ is estimated to be 10^10^ tons, making it an important component of the biogeochemical sulfur cycle [1,2]. Because of its ubiquitous distribution, SQ is expected to be degraded by diverse bacteria in different habitats. In terrestrial environments, SQ from fallen leaves is degraded by soil bacteria, which accelerates the mineralization of organosulfur into sulfate and makes it once again available for absorption by plants. In marine environments, where sulfate is abundant (∼28 mM), the production of SQ and other sulfonates by phototrophs, and their degradation by heterotrophic bacteria, constitutes a major component of the carbon flux in the pelagic ecosystem [3]. In the anoxic distal section of the human intestinal system, degradation of dietary SQ by a consortium of anaerobic bacteria results in conversion of the sulfonate sulfur to H_2_S [4,5], a toxin linked to intestinal barrier dysfunction, gut inflammation and colorectal cancer [6,7].

Apart from phototrophs, SQDG is also present in the *Bacillus coahuilensis*, a bacterium isolated from an inland lake, where the ability to supplement membrane phospholipids with sulfolipids was proposed to arise from adaptation to an environment rich in sulfur but low in phosphorus [8]. SQ is also part of N-linked glycans on the membrane-associated protein complex, the surface (S)-layer protein and the filament subunit of the acidophilic sulfur-oxidizing archaeon *Sulfolobus acidocaldarius* [9]. In all these organisms, synthesis of SQ from sulfite and UDP-glucose is catalyzed by the NAD^+^-dependent enzyme UDP-sulfoquinovose synthase [9,10].

**Figure 1:**
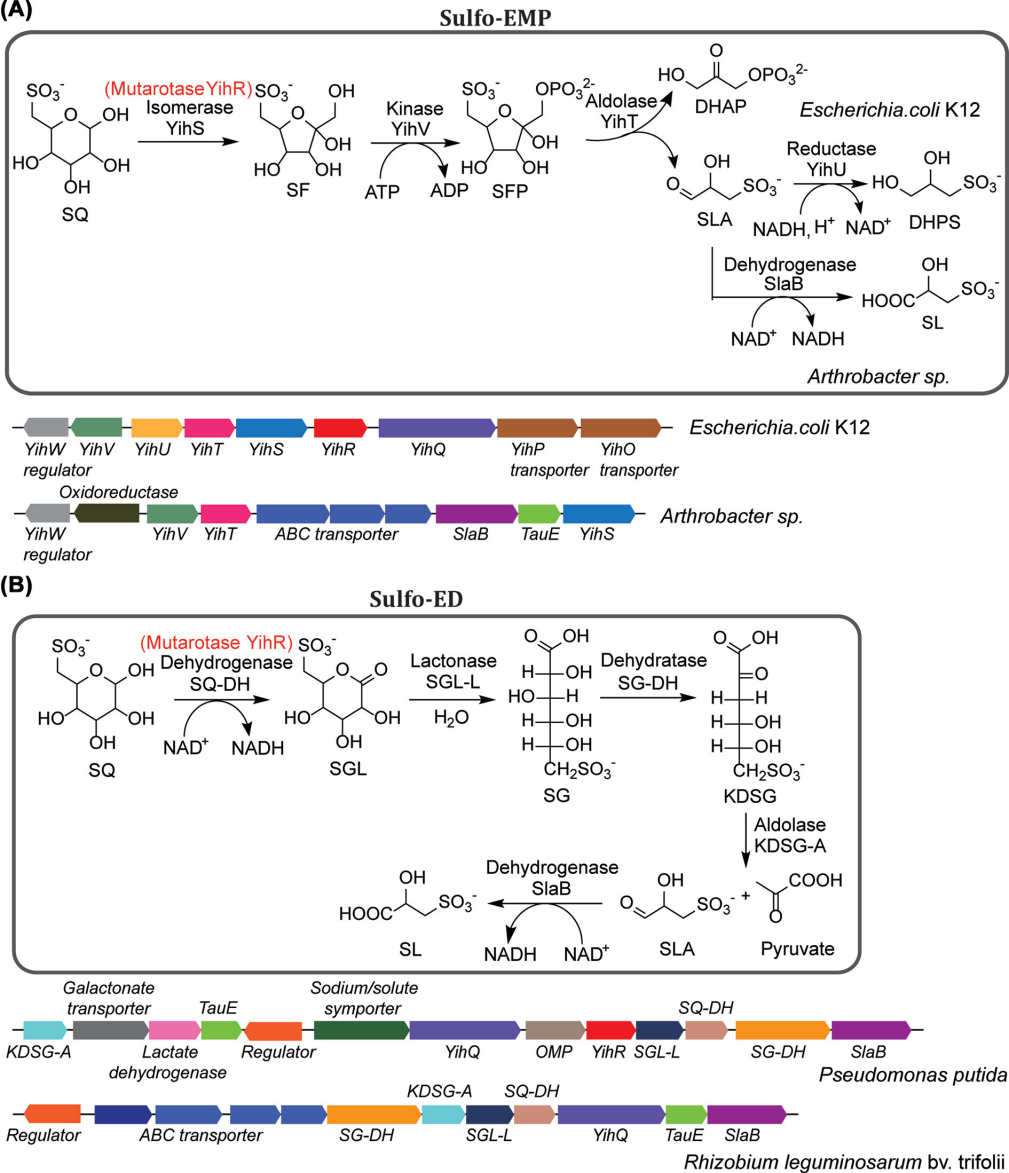
Sulfo-EMP and Sulfo-ED pathways (**A**) Sulfo-EMP pathway with representative gene clusters. (**B**) Sulfo-ED pathway with representative gene clusters. Both pathways contain YihR, a mutarotase labeled in red.

Because of its resemblance to glucose-6-phosphate, degradation of SQ was proposed to proceed through a pathway analogous to glycolysis, referred to as sulfoglycolysis [1,11]. SQ degradation was detected in plants [11] and algae [12,13], but the eukaryotic SQ degradation enzymes remain unknown. Several bacteria, including *Klebsiella* spp. and *Agrobacterium* sp., were isolated for their ability to utilize SQ as a carbon and energy source for growth [1,14]. However, it was only in 2014 that the first sulfoglycolytic pathway was described in molecular detail by Denger et al. [15] who discovered that SQ was utilized by several substrains of *Escherichia coli*, including the sequenced model strain *E. coli* K-12 MG1655. Growth yield on SQ was half that on glucose and was accompanied by release of one equivalent of dihydroxypropanesulfonate (DHPS), suggesting that only half of the SQ carbons are used for growth. The authors demonstrated that the SQ degradation pathway, the sulfo-EMP pathway, is encoded by the inducible *yih* gene cluster and involves isomerization of SQ to 6-deoxy-6-sulfofructose (SF), phosphorylation to SF-1-phosphate (SF-1P), followed by aldol cleavage to dihydroxyacetone phosphate (DHAP) and sulfolactaldehyde (SLA), analogous to the EMP glycolytic pathway. The EMP pathway is widely distributed in cellular organisms from all three domains of life and couples the degradation of glucose to the production of ATP, NADH, and pyruvate. In respiratory organisms, the pyruvate is oxidized to acetyl-CoA and further metabolized through the tricarboxylic acid (TCA) cycle, while in fermenting organisms, the pyruvate is dissimilated through a variety of pathways [16–18]. The *yih* gene cluster involved in sulfo-EMP was noted to be widespread among Gram-negative Enterobacteriaceae bacteria (Figure 1A).

**Figure 2:**
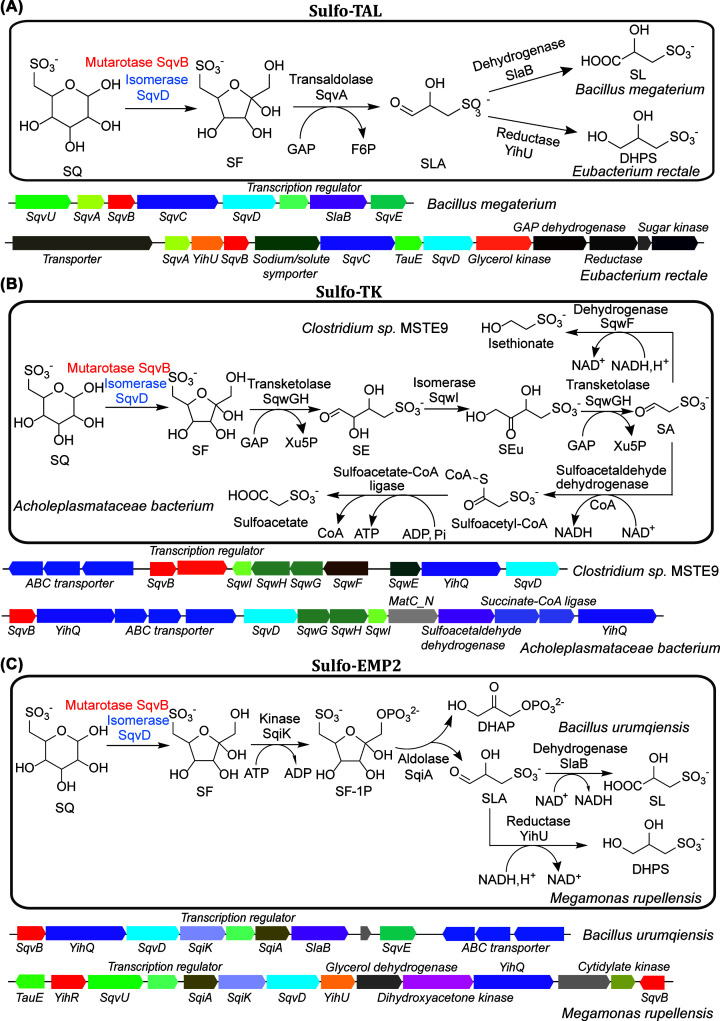
Sulfo-TAL, Sulfo-TK and Sulfo-EMP2 pathways (**A**) Sulfo-TAL pathway with representative gene clusters. (**B**) Sulfo-TK pathway with representative gene clusters. (**C**) Sulfo-EMP2 pathway with representative gene clusters. These three pathways share SqvB, a putative mutarotase labeled in red and an isomerase SqvD labeled in blue.

**Figure 3:**
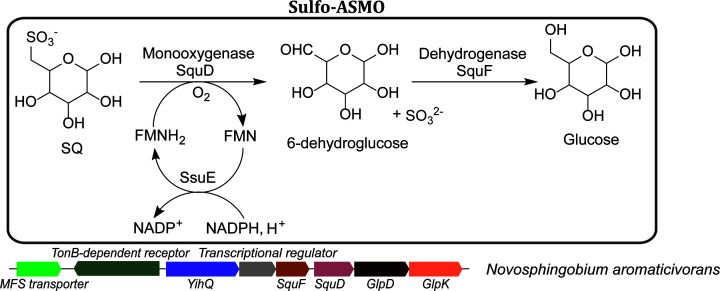
Sulfo-ASMO pathway with a representative gene cluster In the first step of sulfo-ASMO pathway, oxygenolytic C-S cleavage of SQ catalyzed by the flavin-dependent SQ monooxygenase SquD produces sulfite and 6-dehydroglucose. Many of the sulfo-ASMO gene clusters also encode a SsuE homolog that catalyzes the NAD(P)H-dependent regeneration of the reduced flavin cofactor for SquD. For those that lack SsuE, other flavin reductases may be involved (e.g. in *N. aromaticivorans*). In the second step, 6-dehydroglucose is reduced to glucose by the NAD(P)H-dependent glucose-6-dehydrogenase SquF, allowing the complete utilization of SQ carbons.

Given the purported abundance of SQ, it is reasonable to expect the ability to utilize this sulfosugar to be widespread among bacteria inhabiting different environments. Indeed, subsequent research demonstrated that SQ is degraded by phylogenetically and metabolically diverse bacteria, through a variety of biochemical mechanisms. Following the pioneering discovery of sulfoglycolysis in *E. coli*, Felux et al. reported a second sulfoglycolytic pathway in *Pseudomonas putida* SQ1 and related Gram-negative bacteria, which involves oxidation of SQ to 6-deoxy-6-sulfogluconate (SG), analogous to the ED glycolytic pathway (sulfo-ED) (Figure 1B) [19]. Subsequently, Frommeyer et al. and Liu et al. reported a third sulfoglycolytic pathway, present in diverse Gram-positive bacteria, involving cleavage of SF by a transaldolase (sulfo-TAL), related to that in the PP pathway (Figure 2A) [20,21]. Most recently, Liu et al. reported a fourth sulfoglycolytic pathway involving cleavage of SF by a transketolase (sulfo-TK), related to that in the PP pathway (Figure 2B), and a variant of the sulfo-EMP pathway (sulfo-EMP2) in Gram-positive bacteria that uses a non-orthologous set of enzymes (Figure 2C) [22]. In addition to these four sulfoglycolytic pathways, Sharma et al. and Liu et al. reported a non-sulfoglycolytic SQ degradation pathway, involving oxygenolytic C-S cleavage of SQ to form sulfite and 6-dehydroglucose, catalyzed by a flavin-dependent alkanesulfonate monoxygenase (sulfo-ASMO) (Figure 3) [22,23].

Here we review the enzymes and pathways involved in SQ degradation in different bacteria. A recent comprehensive review by Snow et al. includes detailed descriptions of the structures and mechanisms of enzymes in the sulfo-EMP and sulfo-ASMO pathways, pre-sulfoglycolytic enzymes including sulfolipase and sulfoquinovosidase involved in release of SQ from SQDG, and an analysis of the energy balance and carbon flux of the different pathways [24]. Here, we will elaborate on enzymes in sulfo-TAL, sulfo-TK and sulfo-EMP2 pathways, and the fates of the sulfonate by-products.

### Sulfo-EMP pathway

Lipase-catalyzed cleavage of the SQDG fatty acyl groups produces sulfoquinovosyl glycerol (SQGro), which is degraded by enzymes encoded in the *E. coli yih* gene cluster [15]. Hydrolysis of SQGro by sulfoquinovosidase YihQ stereospecifically produces the α-SQ anomer [25], which is isomerized to β-SQ by the SQ mutarotase YihR (PF01263, related to galactose mutarotase) [26]. The slow rate of uncatalyzed SQ mutarotation (with a half-life of ∼300 min) suggests the need for YihR catalysis (Figure 1A) [26]. The β-SQ is then isomerized to SF by the SQ isomerase YihS (PF07221) [27]. YihS is related to enzymes catalyzing aldose-2-epimerization and, in addition to SF, also catalyzes the reversible 2-epimerization of SQ to 6-deoxy-6-sulforhamnose (SR) as a side reaction [27]. SF is then phosphorylated to SF-1P by the SF kinase YihV (PF00294, same family as the *E. coli* phosphofructokinase isozyme PfkB) [27], and then cleaved into DHAP and SLA by SF-1P aldolase YihT (PF01791, same family as the *E. coli* fructose-bisphosphate aldolase isozyme FbaB) (Figure 1A) [27]. The DHAP is metabolized through *E. coli* central metabolic pathways, while the SLA reductase YihU catalyzes NADH-dependent reduction of SLA to DHPS [28], which is then exported.

Degradation of SQ through the sulfo-EMP pathway in *Arthrobacter* spp. and related Actinobacteria was recently reported, involving gene clusters encoding orthologs of YihS, YihV and YihT [29]. These bacteria lack close homologs of YihQ and YihR but are nevertheless able to utilize both free SQ and methyl α-sulfoquinovoside, suggesting the involvement of a non-specific sulfoquinovosidase. In addition, these bacteria lack YihU but instead encode a SLA dehydrogenase, which catalyzes the NAD^+^-dependent oxidation of SLA to sulfolactate (SL) (Figure 1A). It was suggested that the NADH-consuming DHPS formation might be favored by *E. coli* and related facultative anaerobic fermenting bacteria, while the NADH-generating SL formation might be favored by *Arthrobacter* spp. and other aerobic bacteria.

### Sulfo-ED pathway

The sulfo-ED pathway was first studied in the environmental isolate *P. putida* SQ1 [19], and also studied in the plant symbiont *Rhizobium leguminosarum* SRDI565 [30]. In this pathway, α-SQ is oxidized to 6-deoxy-6-sufogluconolactone (SGL) by the NAD^+^-dependent SQ-1-dehydrogenase, belonging to the short chain alcohol dehydrogenase (ADH) family (PF13561, which encompasses diverse enzymes with ADH activity including several aldose-1-dehydrogenases). SGL is hydrolyzed to SG by SGL-lactonase (gluconolactonase family, PF08450) and then dehydrated to 2-keto-3,6-deoxy-6-sulfogluconate (KDSG) by SG dehydratase (PF00920, same family as 6-phosphogluconate dehydratase), and subsequently cleaved into pyruvate and SLA by KDSG aldolase (PF03328, same family as 5-keto-4-deoxy-D-glucarate aldolase) (Figure 1B). The pyruvate is oxidized to acetyl-CoA and further metabolized through the TCA cycle, while the SLA is oxidized to SL and exported. Gene clusters encoding enzymes in the sulfo-ED pathway are present in Gram-negative soil bacteria in the classes α-, β- and γ-proteobacteria (Figure 1B) [19].

### Sulfo-TAL pathway

The sulfo-TAL pathway was studied in Gram-positive Firmicutes bacteria, including the aerobic *Bacillus megaterium* DSM 1804 [21] and *Bacillus aryabhattai*, and the anaerobic intestinal bacteria *Enterococcus gilvus*, *Clostridium symbiosum*, and *Eubacterium rectale* [20]. Like the sulfo-EMP pathway, the sulfo-TAL pathway requires isomerization of SQ to SF but achieves this using a non-orthologous enzyme SqvD (PF02952, same family as *E. coli* Mn^2+^-dependent fucose isomerase FucI), with the possible involvement of putative SQ mutarotase SqvB (IPR024060, a distant homolog of *E. coli* Ni^2+^-dependent ureidoglycolate lyase AllA) [21]. Following its formation, SF is cleaved into SLA and an enzyme-bound glycerone fragment by SF transaldolase SqvA, a close homolog of the transaldolase (PF00923) in the PP pathway [20,21]. Like other transaldolases, SqvA can transfer the glycerone unit to glyceraldehyde phosphate (GAP) to form fructose-6-phosphate (Figure 2A), and also to erythrose-4-phosphate to form sedoheptulose-7-phosphate [20], for subsequent glycolytic degradation. The SLA is oxidized to SL in the aerobic Bacilli or reduced to DHPS in the anaerobic Firmicutes bacteria (Figure 2A). Apart from Firmicutes bacteria, variants of the sulfo-TAL gene cluster are also present in many Thermotogae and Chloroflexi bacteria [21].

### Sulfo-TK pathway

The gene clusters for the sulfo-TK pathway in *Clostridium* sp. MSTE9 (together with sulfo-EMP2 in *Bacillus urumqiensis* and sulfo-ASMO in *Novosphingobium aromaticivorans* DSM 12444) were discovered through a bioinformatics study of gene clusters containing sulfoquinovosidase YihQ, and the constituent enzymes were recombinantly produced and biochemically characterized [22]. The preceding sulfoglycolytic pathways involve cleavage of the sulfo-sugar into two C3 fragments by enzymes through retro-aldol mechanisms, with C1-C3 directed to central metabolism, and C4-C6 forming a sulfonate by-product. By contrast, the sulfo-TK pathway involves sequential cleavage of the sulfo-sugar into three C2 fragments by a thiamine pyrophosphate- (TPP-) dependent transketolase, with C1-4 directed to central metabolism, and C5-6 forming the sulfonate by-product, thus allowing for greater utilization of the SQ carbon atoms (Figure 2B) [22]. In this pathway, SQ is first converted to SF by SqvD (with the possible involvement of SqvB), similar to the sulfo-TAL pathway. SF is then cleaved into 4-deoxy-4-sulfoerythrose (SE) and an enzyme-bound ketol fragment by the TPP-dependent SF transketolase SqwGH, a close homolog of the transketolase (PF02779) in the PP pathway. Like other transketolases, SqwGH can transfer the ketol unit to GAP to form xylulose-5-phosphate, for subsequent degradation through the PP pathway (Figure 2B) [22]. The activity of SqwGH with other ketol acceptors in the PP pathway, such as erythrose-4-phosphate or ribose-5-phosphate, was not tested.

Following its formation, SE is isomerized to 4-deoxy-4-sulfoerythrulose (SEu), proposed to be catalyzed by the putative SE isomerase SqwI (PF02502, same family as galactose-6-phosphate isomerase, ribose-5-phosphate isomerase and erythrulose-4-phosphate isomerase) (Figure 2B). Direct measurement of SqwI activity was hampered by the apparent spontaneous isomerization of SE to SEu, and further biochemical characterization of this enzyme is required. SqwGH then cleaves SEu to form sulfoacetaldehyde, similar to its cleavage of SF. In Clostridia, the sulfoacetaldehyde is reduced to isethionate by the NADH-dependent ADH SqwF (metal-dependent ADH family, PF00465). In other bacteria, the sulfo-TK gene clusters contain homologs of the NAD^+^-dependent sulfoacetaldehyde dehydrogenase and sulfoacetyl-CoA ligase instead of SqwF, suggesting oxidation of sulfoacetaldehyde into sulfoacetate coupled to ADP phosphorylation (Figure 2B) [22].

### Sulfo-EMP2 pathway

Compared with the sulfo-EMP pathway, the sulfo-EMP2 pathway proceeds via identical metabolic intermediates but uses a non-orthologous set of enzymes. In this pathway, SQ is first converted to SF by SqvD (with the possible involvement of SqvB), similar to the sulfo-TAL and sulfo-TK pathways. SF is then phosphorylated to SF-1P by the SF kinase SqiK (PF00365, same family as the *E. coli* phosphofructokinase isozyme PfkA), and then cleaved into DHAP and SLA by SF-1P aldolase SqiA (PF01116, same family as the *E. coli* Zn^2+^-dependent fructose-bisphosphate aldolase isozyme FbaA). The sulfo-EMP2 pathway is present in Gram-positive Firmicutes bacteria. Similar to the sulfo-TAL pathway, the SLA is oxidized to SL in the aerobic Bacilli and reduced to DHPS in the anaerobes like *Megamonas rupellensis* (Figure 2C) [22].

### Comparison of enzymes in the sulfo-EMP and sulfo-EMP2 pathways

**Figure 4:**
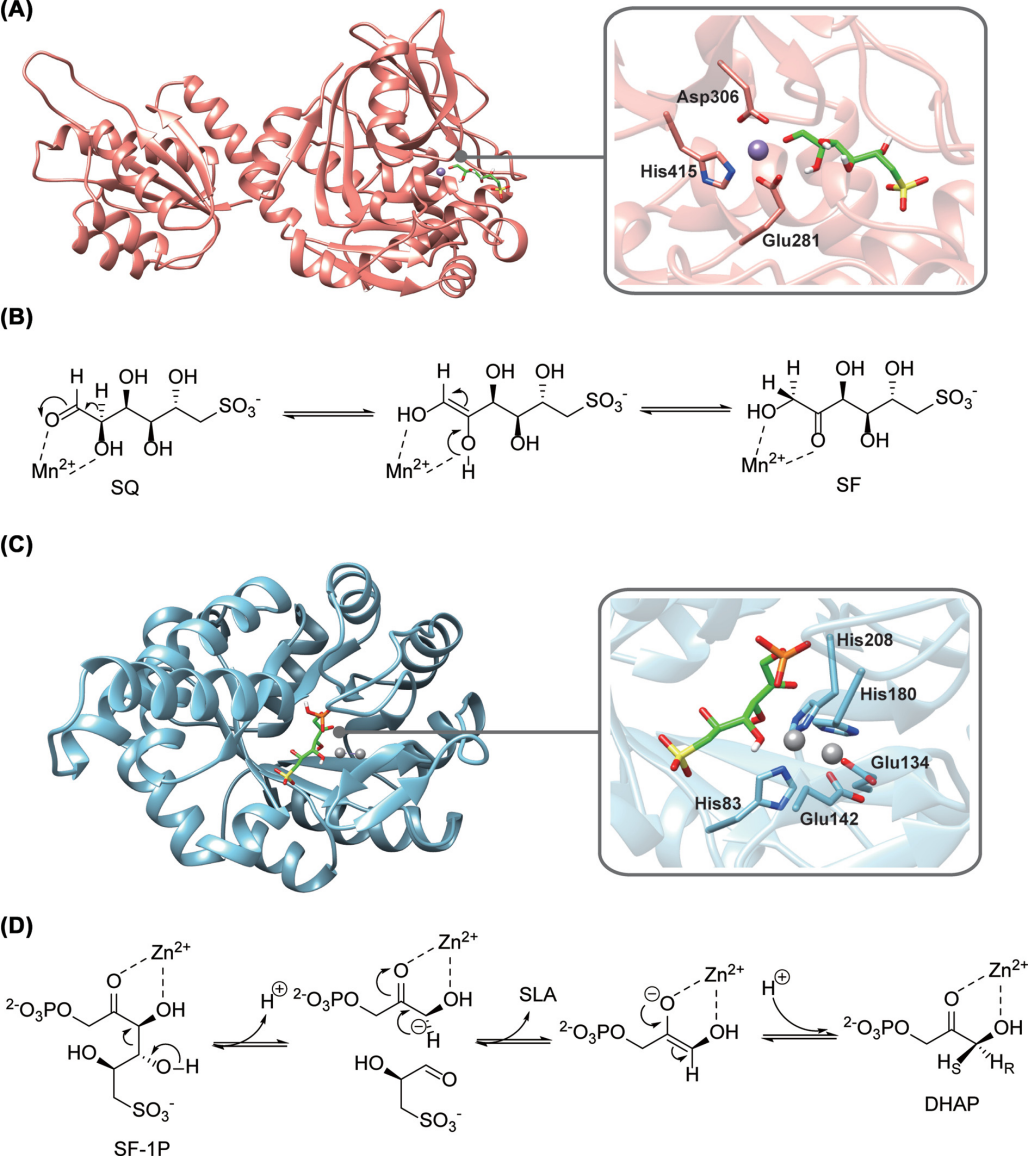
Structural models and proposed catalytic schemes for two key enzymes in the sulfo-EMP2 pathway (**A**) Structural model of SqvD (left) and the zoom-in view of the active site (right), showing conserved Mn^2+^-binding residues. (**B**) Proposed catalytic scheme for SqvD involving an ene-diol intermediate. (**C**) Structural model of SqiA (left) and the zoom-in view of the active site (right), showing conserved Zn^2+^-binding residues. The mobile Zn^2+^ cofactor occupies two mutually exclusive sites. (**D**) Proposed catalytic scheme for SqiA. Both models were constructed using AlphaFold2 [29] in ColabFold [30]. The putative Mn^2+^ site of SqvD and two putative Zn^2+^ sites of SqiA were estimated by alignment with the crystal structures of *E. coli* FucI (PDB: 1FUI) [28] and *Helicobacter pylori* class II fructose-1,6-biphosphate adolase (5UCK) [33], respectively.

The sulfo-EMP enzymes have been reviewed in detail by Snow et al. [21], and here we focus on the functionally equivalent sulfo-EMP2 enzymes (some of which are also involved in sulfo-TAL and sulfo-TK), highlighting the mechanistic differences between the corresponding enzymes in the two pathways. The sulfo-EMP SQ isomerase YihS is a homolog of aldose-2-epimerases [27], while the sulfo-EMP2 SQ isomerase SqvD is a homolog of fucose isomerase FucI. Unlike YihS, which catalyzes interconversion of the pyranose and furanose form of SQ, FucI catalyzes interconversion of open-chain L-fucose and L-fuculose. The FucI catalytic mechanism is thought to involve coordination of the substrate O1 and O2 atoms to an active-site Mn^2+^ cofactor, and to proceed via a Mn^2+^-coordinated ene-diol intermediate [31]. Despite the low sequence homology between *B. urumqiensis* SqvD and *E. coli* FucI (PDB: 1FUI, ∼20% sequence identity) [31], a structural model constructed using AlphaFold2 [32] in ColabFold [33] showed that the Mn^2+^ ligands are conserved (Figure 4A), suggesting a similar catalytic scheme (Figure 4B). Unlike YihS, SqvD is not expected to catalyze SQ 2-epimerization, and the formation of SR was not observed in assays of SqvD [21].

As with the sulfo-EMP pathway, analysis of the sulfo-EMP2 pathway requires careful consideration of SQ ring chain tautomerism. The sulfo-EMP2 gene clusters contain sulfoquinovosidase YihQ, which releases SQ in the α-pyranose form, and the slow rate of uncatalyzed SQ mutarotation suggests a correspondingly slow rate of uncatalyzed conversion of SQ from the pyranose to the open-chain form required by SqvD. Instead of the SQ mutarotase YihR, the majority of sulfo-EMP2 (also sulfo-TAL and sulfo-TK) gene clusters contain the enzyme SqvB (domain of unknown function DUF4867, PF16161). SqvB is a distant homolog of *E. coli* ureidoglycolate lyase AllA, which catalyzes cleavage of ureidoglycolate into urea and glycolate via a mechanism involving an active-site Ni^2+^ cofactor [34,35]. We propose that SqvB catalyzes the mechanistically related cleavage of SQ-pyranose to open-chain SQ, which serves as the substrate of SqvD (this hypothesis is currently under investigation). We note that some variants of the gene clusters for sulfo-EMP and sulfo-EMP2 (described in the next section) contain SqvD in association with YihR instead of SqvB, suggesting that YihR can also catalyze the formation of open-chain SQ, possibly through release of the open-chain mutarotation intermediate.

The SF-1P aldolases of the sulfo-EMP and sulfo-EMP2 pathways also have distinct catalytic mechanisms. The sulfo-EMP aldolase YihT is a homolog of *E. coli* FbaB, while the sulfo-EMP2 aldolase SqiA is a homolog of *E. coli* FbaA. The former is a class I aldolase and uses a catalytic lysine residue [27], while the latter is a class II aldolase, and uses Zn^2+^ in its catalytic mechanism (Figure 4C,D) [36]. The crystal structure of *Helicobacter pylori* class II fructose-1,6-biphosphate adolase (Fba, PDB: 5UCK) contains a mobile Zn^2+^ cofactor occupying two mutually exclusive sites, and it was suggested that turnover may be accompanied by relocation of Zn^2+^ between sites [36]. Its catalytic mechanism is thought to involve coordination of the substrate O2 and O3 atoms to Zn^2+^, followed by aldol cleavage to give a Zn^2+^-coordinated ene-diolate intermediate. An AlphaFold2 [32,33] model of *B. urumqiensis* SqiA (40% sequence identity to *H. pylori* Fba) showed that the ligands for both Zn^2+^ sites are conserved (Figure 4C), suggesting a similar catalytic scheme (Figure 4D).

The distribution of the sulfo-EMP and sulfo-EMP2 gene clusters suggests convergent evolution of this pathway in Gram-negative and Gram-positive bacteria respectively [22]. The functional interchangeability of the corresponding enzymes in the two gene clusters is evidenced by the occurrence of hybrid gene clusters where sulfo-EMP2 isozyme replaces that of sulfo-EMP, e.g., the sulfo-EMP gene cluster in a Yersiniaceae bacterium contains SqvD in place YihS as the SQ isomerase, and SqiA in place of YihT as the SF-1P aldolase [22]. Several of the sulfo-EMP2 enzymes (SqvD, SqiA and putatively SqvB) are dependent on divalent metals, while the respective sulfo-EMP enzymes (YihS, YihT and YihR) are not, suggesting that metal trafficking or bioavailability may have played a role during evolution.

### Sulfo-ASMO pathway

The above sulfoglycolytic pathways involve sulfo-sugar C-C cleavage, but not sulfonate C-S cleavage, and produce organosulfonates as by-products. By contrast, the non-sulfoglycolytic sulfo-ASMO pathway is initiated by SQ sulfonate C-S cleavage and allows the complete utilization of the SQ carbon atoms. This pathway was studied in *Agrobacterium tumefaciens* [23] and in *N. aromaticivorans* [22]. Agrobacterium strains had previously been known to utilize SQ as a substrate for growth [1], but its SQ degradation pathway was only recently reported in an outstanding study by Sharma et al., involving gene cluster identification by differential proteomics, and biochemical and crystallographic characterization of a SQGro ABC transporter and SQ degradation enzymes. The same group also previously reported the biochemical and crystallographic characterization of *A. tumefaciens* sulfoquinovosidase [37]. The *N. aromaticivorans* SQ degradation pathway was found through a contrasting approach by Liu et al., involving gene cluster identification by large scale genome mining, and biochemical characterization of recombinant enzymes. In this pathway, SQ undergoes oxygenolytic C-S cleavage into sulfite and 6-dehydroglucose, catalyzed by the SQ monooxygenase SquD, a close homolog of the *E. coli* flavin-dependent alkanesulfonate monooxygenase SsuD. In the SsuDE two-component monooxygenase system, SsuE catalyzes the NAD(P)H-dependent reduction of a flavin (FMN or FAD) cofactor, which is transferred to SsuD for the O_2_ activation. Many of the sulfo-ASMO gene clusters contain a close homolog of SsuE. For those that lack SsuE (e.g. in *N. aromaticivorans*), generation of the reduced flavin required by SquD is presumed to be catalyzed by other flavin reductases in the cell. The SquD product 6-dehydroglucose is reduced to glucose by the NAD(P)H-dependent glucose-6-dehydrogenase SquF (aldo-keto reductase family, PF00248) (Figure 3). Because of its requirement for O_2_, sulfo-ASMO gene clusters are only present in aerobic bacteria, largely α- and β-proteobacteria [22,23].

### Extended sulfoglycolytic pathways

**Figure 5:**
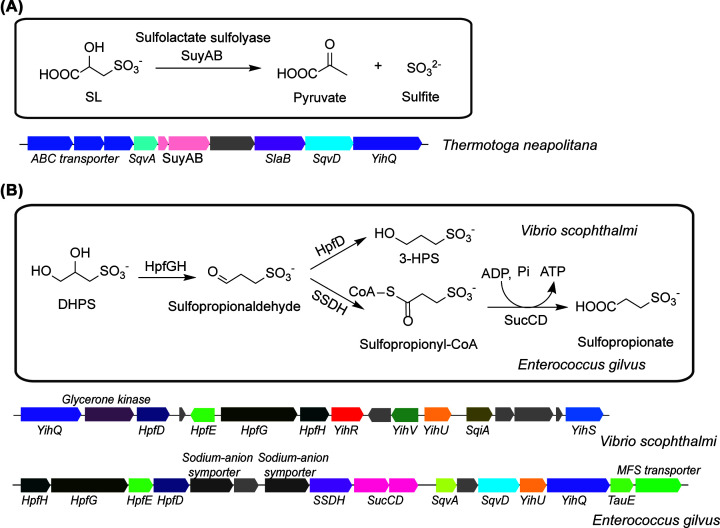
Extended sulfoglycolysis pathways (**A**) Extended sulfoglycolysis pathway showing the fate of SL with a representative gene cluster. (**B**) Extended sulfoglycolysis pathway showing the fate of DHPS with representative gene clusters.

Some bacteria contain gene clusters for ‘extended’ sulfoglycolytic pathways, which include genes for further metabolism of the sulfonate by-products. For example, the sulfo-TAL gene clusters in Thermotogae and Chloroflexi bacteria contains *(R)-*sulfolactate sulfolyase SuyAB (galactarate dehydratase family, PF04295), which catalyzes C-S cleavage to form sulfite and pyruvate, suggesting a mechanism for the complete utilization of the SQ carbon atoms in these bacteria (Figure 5A) [21]. The sulfo-EMP gene cluster in *Vibrio scophthalmi* and sulfo-TAL gene cluster in *Enterococcus glivus* contain the O_2_-sensitive DHPS dehydratase HpfG (glycyl radical enzyme family, PF02901) and NADH-dependent 3-sulfopropionaldehyde reductase HpfD (metal-dependent ADH family, PF00465), suggesting NADH-consuming net reduction of DHPS to 3-sulfopropanol during anaerobic fermentative growth (Figure 5B) [38]. In addition, the gene cluster in *E. gilvus* also contains a putative NAD^+^- and CoA-dependent 3-sulfopropionaldehyde dehydrogenase (PF00171, related to succinic semialdehyde dehydrogenase) and ATP-dependent 3-sulfopropionyl-CoA synthetase (PF00549, related to succinyl-CoA synthetase), suggesting an alternative mechanism of sulfopropionaldehyde degradation involving oxidation to sulfopropionate coupled to ADP phosphorylation (Figure 5B).

### Metabolism of sulfonate by-products

With the exception of the sulfo-SMO pathway, the other SQ degradation pathways do not involve cleavage of the inert sulfonate C-S bond and are accompanied by formation of a sulfonate by-product. The sulfo-EMP(2), sulfo-ED and sulfo-TAL pathways produce a C3 sulfonate by-product (DHPS or SL), while the sulfo-TK pathway produces a C2 sulfonate by-product (isethionate or sulfoacetate). Thus, the complete degradation of SQ through these pathways, including mineralization of the sulfonate sulfur, requires the involvement of other bacteria capable of sulfonate C-S cleavage. Enzymes and biochemical pathways for degradation of C2 and C3 sulfonates have been extensively studied, particularly in aerobic bacteria, as reviewed by Cook et al. [39,40]. Several recent studies have also clarified the mechanism of sulfonate degradation in strict anaerobic bacteria, including sulfate- and sulfite-reducing bacteria (SSRB), which utilize sulfonate-derived sulfite as a terminal electron acceptor (TEA) for anaerobic respiration, reducing it to H_2_S [4,38,41,42].

Insights into possible mechanisms of SQ sulfur mineralization by communities of environmental bacteria were provided by Denger et al. [14], who reported two examples of complete degradation of SQ by defined bacterial co-culture systems. In the first, SQ is metabolized via the sulfo-ED pathway by *P. putida* SQ1 (isolated from littoral sediment), and the resultant (*S*)*-*SL is degraded by *Paracoccus pantotrophus* NKNCYSA (from anaerobic sludge) [43,44]. In the second example, SQ is degraded via the sulfo-EMP pathway by *Klebsiella oxytoca* TauN1 (from soil) [45], and the resultant (*S*)*-*DHPS degraded by *Cupriavidus pinatubonensis* JMP134 (from volcanic mudflow deposits) [46]. Of these four strains, *P. putida* SQ1 was isolated from enrichment cultures with SQ as the sole carbon source, while the other three were previously studied for their ability to degrade various sulfonates. Crucially, neither *P. pantotrophus* nor *C. pinatubonensis* were able to metabolize SQ, but they were able to utilize the respective C3 sulfonates from spent media as substrates for growth, converting the sulfonate sulfur into a stoichiometric amount of sulfate [14].

In both *P. pantotrophus* and *C. pinatubonensis*, sulfonate C-S cleavage is ultimately catalyzed by (*R*)*-*sulfolactate sulfolyase SuyAB. In most sequenced *Paracoccus* species (e.g., *P. alkanivorans*), the genome neighborhood of SuyAB contains close homologs of (*S*)-sulfolactate dehydrogenase SlcC and (*R*)-sulfolactate dehydrogenase ComC, which have been studied in *Chromohalobacter salexigens*, where they were proposed to catalyze racemization of (*S*)-SL prior to desulfonation by SuyAB [47]. In *C. pinatubonensis* JMP134, (*S*)*-*DHPS dehydrogenase HpsO and (*R*)*-*DHPS dehydrogenase HpsP catalyze racemization of (*S*)-DHPS, followed by oxidation by (*R*)-DHPS dehydrogenase HpsN to form (*R*)-SL (in two steps via SLA), and desulfonation by SuyAB [43]. In both bacteria, sulfite released by SuyAB is oxidized to sulfate by the molybdenum-dependent sulfite dehydrogenase, with cytochrome *C* as an electron acceptor [48].

Bacterial degradation of SQ and other sulfonates in anoxic environments often terminates in the formation of H_2_S instead of sulfate, due to the involvement of SSRB. This process is of great relevance to sulfur metabolism and H_2_S production in the human gut. Burrichter et al. reported a defined bacterial co-culture system for the anaerobic degradation of SQ by *E. coli* K-12 and *Desulfovibrio* sp. strain DF1, isolated from enrichment cultures of anaerobic sewage sludge with DHPS as the sole carbon source and TEA [4]. In this anaerobic system, *E. coli* K-12 ferments SQ via the sulfo-EMP pathway, producing DHPS, and with the resultant GAP metabolized via mixed acid fermentation. In *Desulfovibrio* sp. DF1, DHPS is oxidized to SLA by NAD^+^-dependent DHPS dehydrogenase DhpA (a homolog of *E. coli* SLA reductase YihU), followed by oxidation to SL by NAD^+^-dependent SLA dehydrogenase SlaB (a homolog of SLA dehydrogenase present in other sulfoglycolysis pathways) and desulfonation by SuyAB. Sulfite released by SuyAB is reduced to H_2_S by dissimilatory sulfite reductase (Dsr). Homologs of SuyAB were found in many other SSRB in sequence databases, suggesting the widespread ability for C3 sulfonate degradation in these anaerobic bacteria [4].

Of particular interest is the sulfite reducing bacterium *Bilophila wadsworthia*, a human intestinal pathogen associated with many disease conditions [49,50]. *Bilophila wadsworthia* is unable to utilize sulfate as a TEA, but it is able to degrade a broad range of sulfonates, and to use the resultant sulfite as a TEA. In *B. wadsworthia*, degradation of SL is proposed to involve SuyAB [4], while degradation of DHPS and isethionate (a product of the sulfo-TK pathway) involves O_2_-sensitive enzymes DHPS sulfolyase HpsG [38] and isethionate sulfolyase IseG [41] (glycyl radical enzyme family, PF02901), which catalyze C-S cleavage through a free radical-dependent mechanism. A detailed study by Hanson et al. [5] demonstrated the complete degradation of SQ by a co-culture of *B. wadsworthia* with the abundant human gut bacterium *E. rectale*, in which *E. rectale* converts SQ to DHPS via the sulfo-TAL pathway, and *B. wadsworthia* converts DHPS to H_2_S via HpsG. Analysis of human fecal metatranscriptome datasets suggests the relevance of this pathway in human subjects across various health states.

## Conclusions

Since the initial hypothesis of a glycolytic pathway for SQ degradation in 1963, a total of four sulfoglycolytic pathways have been identified to date (sulfo-EMP(2), sulfo-ED, sulfo-TAL and sulfo-TK), with strong analogies to known glycolytic pathways. Among the naturally occurring sugars, SQ is unique in possessing an anionic and hydrolytically inert sulfonate group, which requires specialized enzymes for C-S cleavage. As a result, most organisms only metabolize a fraction of the SQ carbons and export the remaining carbons as a sulfonate by-product. By contrast, one non-sulfoglycolytic pathway has also been identified, involving oxygenolytic SQ desulfonation (sulfo-ASMO) to release sulfite as a by-product, enabling the complete utilization of SQ carbons (Figure 3).

Further degradation of sulfoglycolysis sulfonate by-products by other bacteria is required for mineralization and recycling of the sulfonate sulfur. Bacterial metabolism of (*S*)-DHPS and (*S*)-SL (products of sulfo-EMP(2), sulfo-ED and sulfo-TAL pathways) and isethionate (a product of the sulfo-TK pathway) are well studied. However, degradation of 3-sulfopropanol and 3-sulfopropionate (products of HpfG-dependent extended sulfoglycolysis pathways in anaerobes) and sulfoacetate (a product of the sulfo-TK pathway) require further investigation, particularly in anaerobic bacterial communities.

While the traditional approach to identification of new SQ degradation pathways has involved isolation and cultivation of SQ-degrading bacteria [1,15,19,20,29], recent research has relied heavily on bioinformatics analyses [20–22]. Several of the pathways (sulfo-TAL, sulfo-EMP2 and sulfo-TK) share enzymes in common (SqvB, SqvD), and the two non-orthologous sulfo-EMP(2) pathways contain isozymes that are frequently interchanged in different organisms [22]. In addition, many of the gene clusters contain characteristic sulfosugar-associated genes, such as the sulfoquinovosidase YihQ [25], the sulfosugar-responsive transcription factor CsqR [27] and various transporters. This interconnectedness has made SQ degradation an excellent model system for the development and application of bioinformatics techniques to explore new enzymes [22]. Further analysis of genomic databases promises to uncover yet more mechanisms for degradation of SQ and related sulfosugars by metabolically diverse bacteria, and to deepen our understanding of bacterial carbohydrate and sulfonate metabolism.

## Abbreviations

ADH: alcohol dehydrogenase
ASMO: alkanesulfonate monooxygenase
DHAP: dihydroxyacetone phosphate
DHPS: dihydroxypropanesulfonate
ED: Entner–Doudoroff
EMP: Embden–Meyerhof–Parnas
GAP: glyceraldehyde phosphate
KDSG: 2-keto-3,6-deoxy-6-sulfogluconate
PP: pentose phosphate
SE: 4-deoxy-4-sulfoerythrose
SEu: 4-deoxy-4-sulfoerythrulose
SF: 6-deoxy-6-sulfofructose
SF-1P: SF-1-phosphate
SG: 6-deoxy-6-sulfogluconate
SGL: 6-deoxy-6-sufogluconolactone
SL: sulfolactate
SLA: sulfolactaldehyde
SQ: sulfoquinovose
SQGro: sulfoquinovosyl glycerol
SR: 6-deoxy-6-sulforhamnose
SSRB: sulfate- and sulfite-reducing bacteria
TEA: terminal electron acceptor
TPP: thiamine pyrophosphate
